# Treatment with aripiprazole once-monthly injectable formulation is effective in improving symptoms and global functioning in schizophrenia with and without comorbid substance use – a post hoc analysis of the ReLiAM study

**DOI:** 10.1186/s12888-022-04397-x

**Published:** 2022-12-08

**Authors:** Howard C. Margolese, Matthieu Boucher, Francois Therrien, Guerline Clerzius

**Affiliations:** 1 Schizophrenia Program, McGill University Health Centre, Montreal, QC Canada; 2 Department of Psychiatry, McGill University, Montreal, QC Canada; 3 MUHC, Allan Memorial Institute, 1025 Pine Ave W, Montreal, QC H3A 1A1 Canada; 4 Medical Affairs, Otsuka Canada Pharmaceutical Inc, St-Laurent, QC Canada; 5 Department of Pharmacology and Therapeutics, Faculty of Medicine and Health Sciences, McGill University, Montreal, QC Canada; 6 Lundbeck Canada Inc., St-Laurent, QC Canada

**Keywords:** Aripiprazole, Injectable, Monthly, Schizophrenia, Substance use

## Abstract

**Background:**

ReLiAM, Real-Life Assessment of Abilify Maintena, was the first reported long-term prospective non-interventional study for patients with schizophrenia treated with aripiprazole once-monthly injectable formulation (AOM) under real-life conditions. ReLiAM’s primary aim was to evaluate the evolution of global functional status in patients treated with AOM for 12 months in Canada.

**Methods:**

The objective of this post hoc analysis of the ReLiAM study is to investigate the treatment effects of real-life use of AOM over a 1-year period in the subgroup of patients with reported substance use compared with patients without substance use.

**Results:**

The results of this post hoc analysis demonstrate that treatment with AOM for 12 months in patients with schizophrenia was comparably effective in improving global functioning in subgroups of patients with and without concomitant substance use.

**Conclusions:**

These results support the use of AOM for the treatment of schizophrenia in patients with or without concomitant substance use.

**Trial registration:**

ClinicalTrials.gov NCT02131415, first posted on May 6, 2014. Overall trial status: Terminated.

**Supplementary Information:**

The online version contains supplementary material available at 10.1186/s12888-022-04397-x.

## Background

Psychoactive substances are frequently used and often abused by patients with schizophrenia [1]. The incidence of substance use disorder (SUD) in patients with schizophrenia is high compared to the average rates of SUD in the general adult population [2]. Nearly half of patients with schizophrenia will develop a SUD in their lifetime, with the most frequently used substances being alcohol and cannabis, at approximately three times the rate of people without schizophrenia [3]. In a study of individuals with severe psychotic disorders, the risk of heavy alcohol use was four times greater compared to the general population [4]. A recent meta-analysis of patients with schizophrenia found a lifetime prevalence of alcohol and cannabis use of 24.3% and 26.2%, respectively [5]. Margolese et al. (2006) reported alcohol (35.6%) and cannabis (35.1%) as the most common primary substances of abuse in a 12-month prospective study of patients with comorbid schizophrenia-spectrum disorders and substance abuse [6]. Data suggest that genetic predisposition to schizophrenia is associated with substance use patterns later in life. In a prospective study, schizophrenia vulnerability was associated with stronger cannabis use during adolescence (age 16–20 years) [7], and previous reports on individuals at genetic risk for developing schizophrenia suggest that cannabis use is an independent risk factor for psychosis [8–10]. Ultimately, the co-occurrence of schizophrenia and SUD is associated with poorer clinical outcomes due to increased symptom severity, psychosocial instability, and treatment non-adherence [3].

The impact of treatment with oral aripiprazole in patients with schizophrenia and comorbid alcohol, cannabis, and stimulant use has been documented [11–13]. A switch from oral to long-acting injectable (LAI) antipsychotic medication is a common strategy to overcome non-adherence in patients with schizophrenia [14, 15]. Recent studies looked at the effect of LAI second-generation antipsychotic medications in various populations with comorbid substance use. Patients with schizophrenia and alcohol use disorder treated with LAI versus oral risperidone had significantly fewer heavy drinking days over time and fewer drinking days per week [16]. Furthermore, patients who took the LAI formulation had better overall treatment adherence. In patients with first-episode psychosis and SUD, a Canadian study found that first-line treatment with LAI compared to oral antipsychotics resulted in a significantly lower relapse rate (67.7% vs. 76.7%) and longer relapse-free survival time (694 vs. 447 days), as well as trends for reduced re-hospitalization rates (48.4% vs. 57.3%) and hospitalization-free survival time (813 vs. 619 days) [17].

ReLiAM, Real-Life Assessment of Abilify Maintena, was the first reported long-term prospective non-interventional study for patients with schizophrenia treated with aripiprazole once-monthly injectable formulation (AOM) under real-life conditions [18]. The primary aim of the ReLiAM study was to evaluate the evolution of global functional status in patients treated with AOM for 12 months in Canada. The primary and secondary outcome measures of global, social, and occupational functioning showed significant improvements with progress from serious to mild impairment in patients with schizophrenia treated with AOM over 1 year. In addition, the severity of illness was significantly reduced. Given the naturalistic design of the ReLiAM study, a subpopulation of patients enrolled had reported substance use. The objective of this *post hoc* analysis is to investigate the treatment effects of real-life use of AOM over a 1-year period in the subgroup of patients with reported substance use compared to patients without substance use.

## Method

### Design

This is a *post hoc* analysis of data from the ReLiAM study. The design of the ReLiAM study, which was a naturalistic, non-interventional, prospective cohort study, was previously described [18]. Briefly, the primary outcome of global functioning was assessed with the Global Assessment of Functioning Scale (GAF) at 3-month intervals for 1 year. The Social and Occupational Functioning Scale (SOFAS), the Clinical Global Impression-Severity Scale (CGI-S), and the Brief Psychiatric Rating Scale (BPRS) were used as secondary outcome measures. Adverse drug reactions (ADR) were also collected. This manuscript adheres to CONSORT guidelines.

### Study participants

Patients were recruited to the ReLiAM study from 17 Canadian community- or hospital-based clinical settings, selected as a representative sample of practice of Canadian psychiatrists who treat patients with schizophrenia spectrum disorders [18]. One hundred and ninety-nine patients were enrolled in the study according to the following inclusion and exclusion criteria:

- Inclusion criteria: Patients diagnosed with schizophrenia, at least mildly ill (CGI-S score of ≥ 3), age 18 years (19 for patients from British Columbia) or older, fluent in English and/or French, able to provide informed consent, and for whom the treating psychiatrist had decided, prior to and independently of enrolment in the study, to prescribe AOM for the treatment of schizophrenia.
- Exclusion criteria: Patients who did not comprehend the informed consent, had contraindications to the use of AOM as specified in the Canadian Product Monograph, had previously received one or more doses of AOM, presented a significant suicidal risk as judged by the investigator, or pregnant or lactating females.

Of those 199 patients who met eligibility criteria, 30 were not included in the analyses due to missing information (1) or the lack of post-baseline assessment (29). Thus, 169 patients constituted the primary analysis population of the ReLiAM study. All patients that received at least 1 dose of AOM were included in the safety assessments.

This *post hoc* analysis reports on changes in primary and secondary outcomes for patients in the ReLiAM study who reported at baseline reported substance use at baseline compared to those who had reported no use. Substance users were defined as patients who, at the baseline visit, reported substance use in the previous 30 days. Non-users were patients who, at the baseline visit, reported no substance use in the previous 30 days. Substance use was based solely on self-report, and any use prior to the 30 days immediately preceding study enrollment was not captured. Heavy alcohol use was defined as ≥7 drinks per week for women and ≥14 drinks per week for men, reported at baseline. Non-heavy users of alcohol included non-users of alcohol. Heavy cannabis use referred to ≥7 marijuana cigarettes per week, reported at baseline, and non-heavy users of cannabis included non-users of cannabis.

### Statistical analysis

Descriptive statistics were produced for all study variables including the mean and standard deviation (SD) for continuous scale variables and frequency distributions for categorical scale variables. All statistical tests were 2-sided and a *p*-value of 0.05 or less was considered significant.

## Results

At baseline, 116 (68.6%) of the 169 eligible patients were active substance users (Table 1). The majority of substance users were male (77.6%). Substances reported by users included nicotine, alcohol, cannabis, and other substances (Table 2). Seventy-four (43.8%) patients used alcohol and 35 patients (20.7%) used cannabis at baseline. In patients who were users of the respective substances at baseline, there was no significant change in reported use of the substance during treatment with AOM.

**Table 1:** Baseline Demographics by Use of Any Substance (yes vs. no) primary analysis set

		**Any Substance Use**		
**Parameter**		Yes (*n* = 116)	No (*n* = 53)	Overall (*n* = 169)
Age at Consent (years) ^a^	Valid n	116	53	169
	Mean [SD (95% CI)]	31.4 [11.47 (29.4, 33.6)]	35.9[13.20 (32.3, 39.6)	32.9[12.18 (31.0, 34.7)
	Min, max	18, 67	18, 69	18, 69
Gender, n (%)	Male	90 (77.6)	24 (45.3)	114 (67.5)
	Female	26 (22.4)	29 (54.7)	55 (32.5)
Race, n (%)	Caucasian	98 (84.5)	31 (58.5)	129 (76.3)
	Black	9 (7.8)	13 (24.5)	22 (13.0)
	Asian	3 (2.6)	5 (9.4)	8 (4.7)
	Other	6 (5.2)	4 (7.5)	10 (5.9)
Region, n (%)	British Columbia	10 (8.6)	1 (1.9)	11 (6.5)
	Prairies^1^	19 (16.4)	11 (20.8)	30 (17.8)
	Ontario	31 (26.7	17 (32.1)	48 (28.4)
	Quebec	51 (44.0)	24 (45.3)	75 (44.4)
	Eastern^2^	5 (4.3)		5 (3.0)

^1^Prairies = Alberta, Saskatchewan, and Manitoba

^2^Eastern = New Brunswick, Newfoundland, Nova Scotia and Prince Edward Island

^a^Age at consent (years) = int[(ICF signed date)-Date of Birth + 1)/365.25]

**Table 2:** Substances Reported by Substance Users

• Nicotine
• Alcohol
• Cannabis
• Cough medicine with codeine
• Cocaine
• 3,4-Methylenedioxy​methamphetamine (MDMA)
• Morning glory seeds
• Energy drinks
• Amphetamines
• γ-hydroxybutyrate (GHB)
• Methamphetamines
• Opioids
• Benzodiazepines

### Primary outcome: GAF

**Fig. 1:**
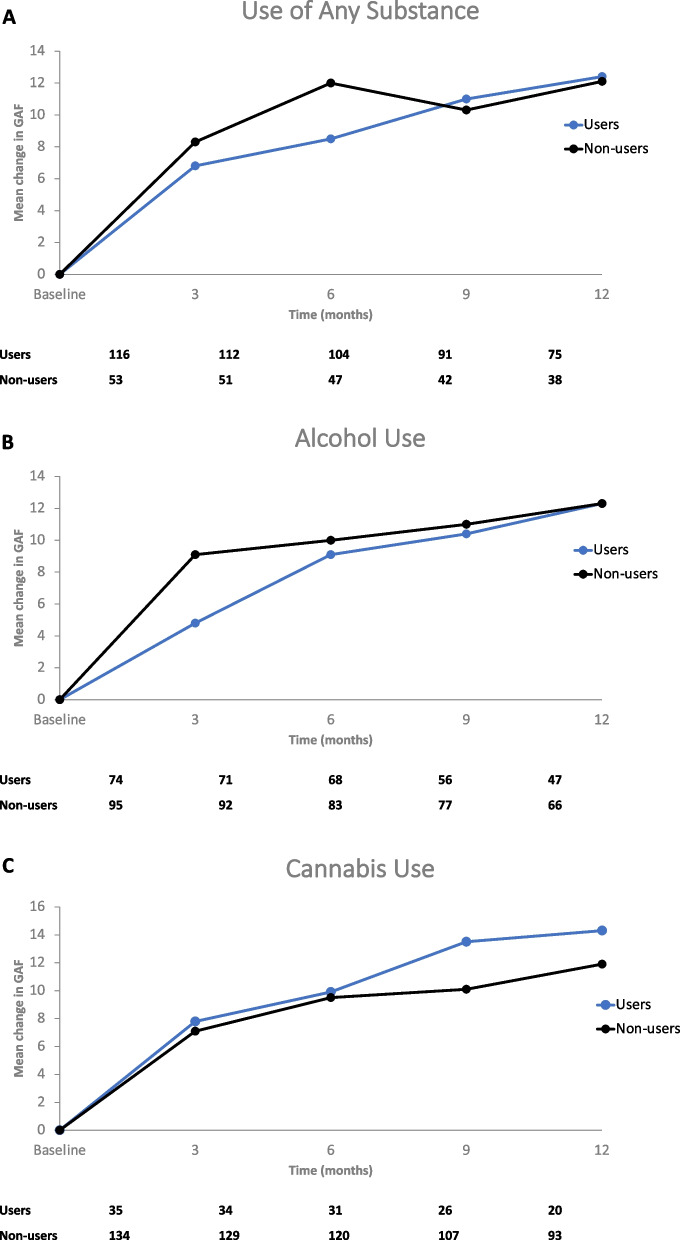
Absolute Change from Baseline in Global Assessment of Functioning Scale (GAF) over Time in Substance Users and Non-Users. **A** Patients reporting any substance use at baseline. **B** Patients reporting alcohol use at baseline. **C** Patients reporting cannabis use at baseline

Overall, improvements in GAF scores after AOM treatment for 12 months were not significantly different between users and non-users of substances, nor between groups of heavy users and non-users. The absolute increase in GAF scores from baseline to 12 months was 12.4 in users of any substances. This was not significantly different from the change in GAF scores observed in non-users of any substances (12.1 change from baseline to 12 months, *P* = 0.939) (Fig. 1A). More specifically, there was no difference in the absolute change in GAF scores from baseline to 12 months between alcohol users and non-users (12.3 vs. 12.3, *P* = 0.988) (Fig. 1B). Cannabis users had a global functioning score improvement of 14.3 from baseline to 12 months, which was not statistically different from non-users of cannabis (11.9, *P* = 0.543) (Fig. 1C).

Although the number of patients in the subgroups were limited, differences in the absolute change in GAF scores over 12 months were similar in both heavy cannabis users and non-heavy cannabis users (absolute change in GAF score from baseline to 12 months: 18.9 vs. 11.8, *P* = 0.222) (Supplemental Fig. 1A). Similar results were observed in heavy alcohol drinkers vs. non-heavy alcohol drinkers, even when considering baseline alcohol users only (absolute change in GAF score from baseline to 12 months: 15.0 vs. 12.1, *P* = 0.641) (Supplemental Fig 1B).

### Secondary outcomes: SOFAS, CGI-S, BPRS

**Fig. 2:**
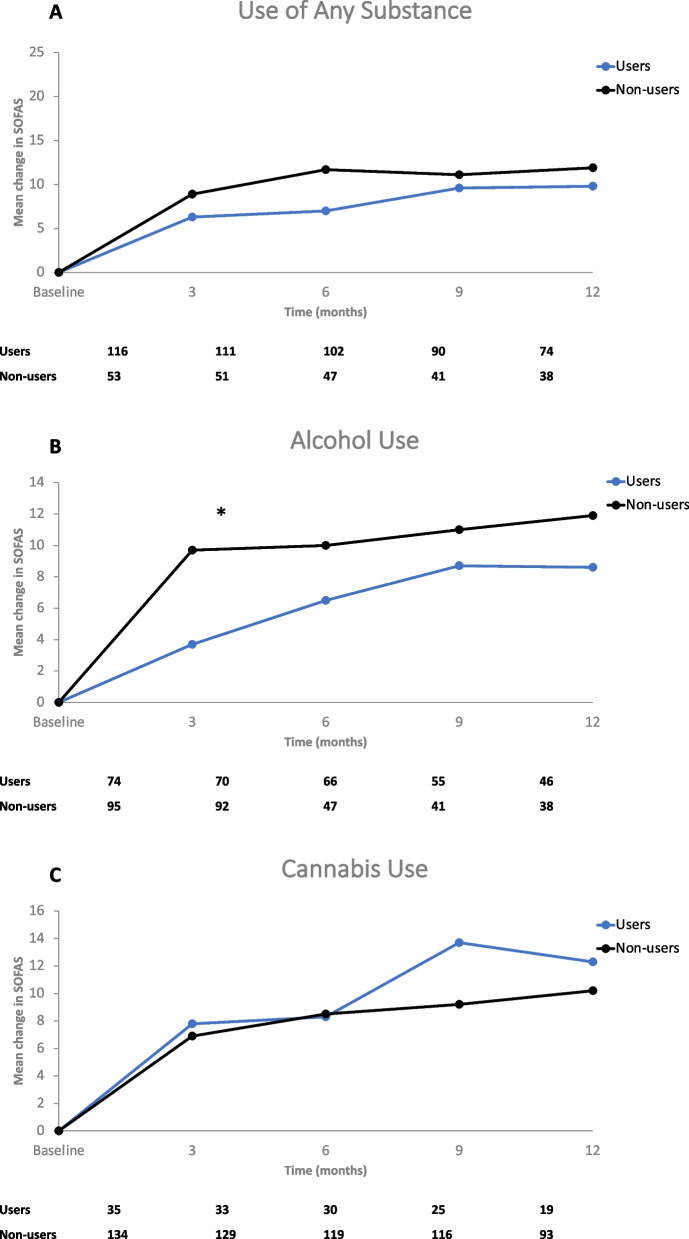
Change in Social and Occupational Functioning Assessment Scale (SOFAS) over Time in Substance Users and Non-Users. **A** Patients reporting any substance use at baseline. **B** Patients reporting alcohol use at baseline. **C** Patients reporting cannabis use at baseline

After AOM treatment for 12 months, there were no statistically significant differences in improvements in social and occupational functioning assessment scale (SOFAS) scores between users and non-users of any substance (respective mean changes from baseline in SOFAS scores were 9.8 vs. 11.9; *P* = 0.424) (Fig. 2A). Although a significant difference between alcohol users and non-users was observed at 3 months (3.7 vs. 9.7; *P* = 0.001), there was no difference in the mean change in SOFAS scores from baseline to 12 months between alcohol users and non-users (8.6 vs. 11.9, *P* = 0.159) (Fig. 2B). Amongst cannabis users and non-users at 12 months, there was no difference in change from baseline SOFAS scores (12.3 vs. 10.2, *P* = 0.501) (Fig. 2C).

**Fig. 3:**
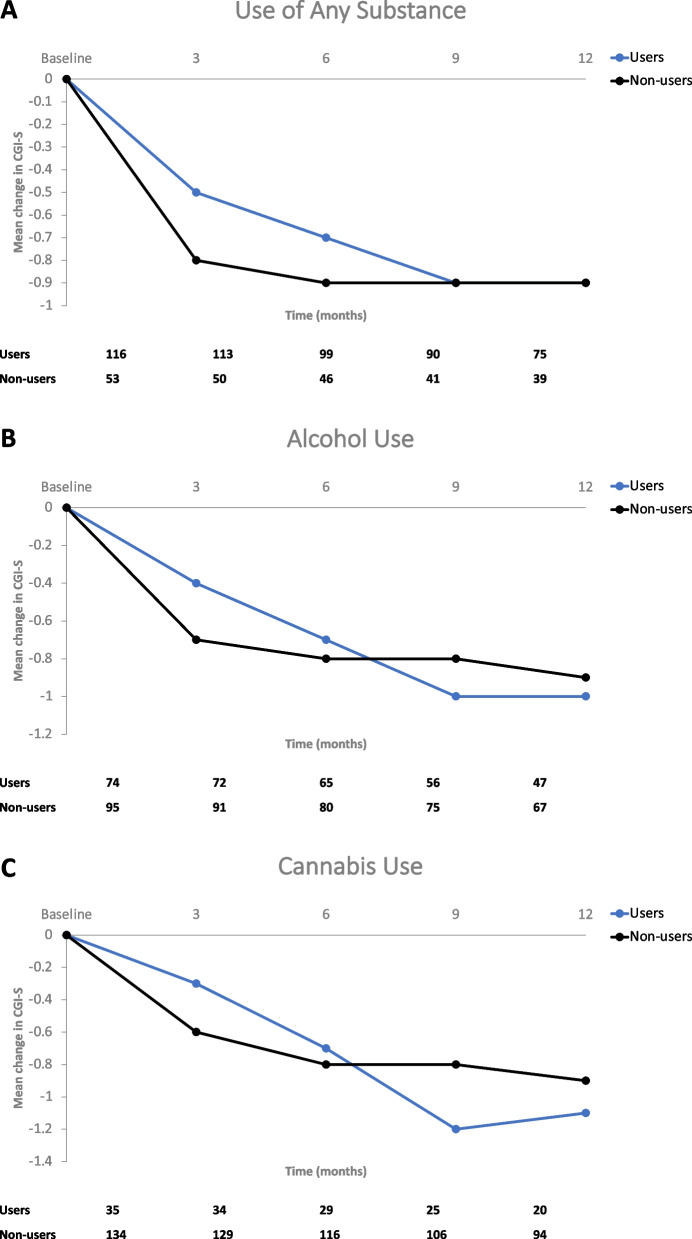
Change in Clinical Global Impression Scale – Severity (CGI-S) over Time in Substance Users and Non-Users. **A** Patients reporting any substance use at baseline. **B** Patients reporting alcohol use at baseline. **C** Patients reporting cannabis use at baseline

Similarly, CGI-S scores showed no difference in mean change from baseline to 12 months between users and non-users of any substance (-0.9 vs. -0.9, *P* = 0.9127) (Fig. 3A). There was also no significant difference in the mean change in CGI-S scores from baseline to 12 months between users and non-users of alcohol (-1.0 vs. -0.9, *P* = 0.6144) (Fig. 3B), or between users and non-users of cannabis (-1.1 vs. -0.9, *P* = 0.4655) (Fig. 3C).

**Fig. 4:**
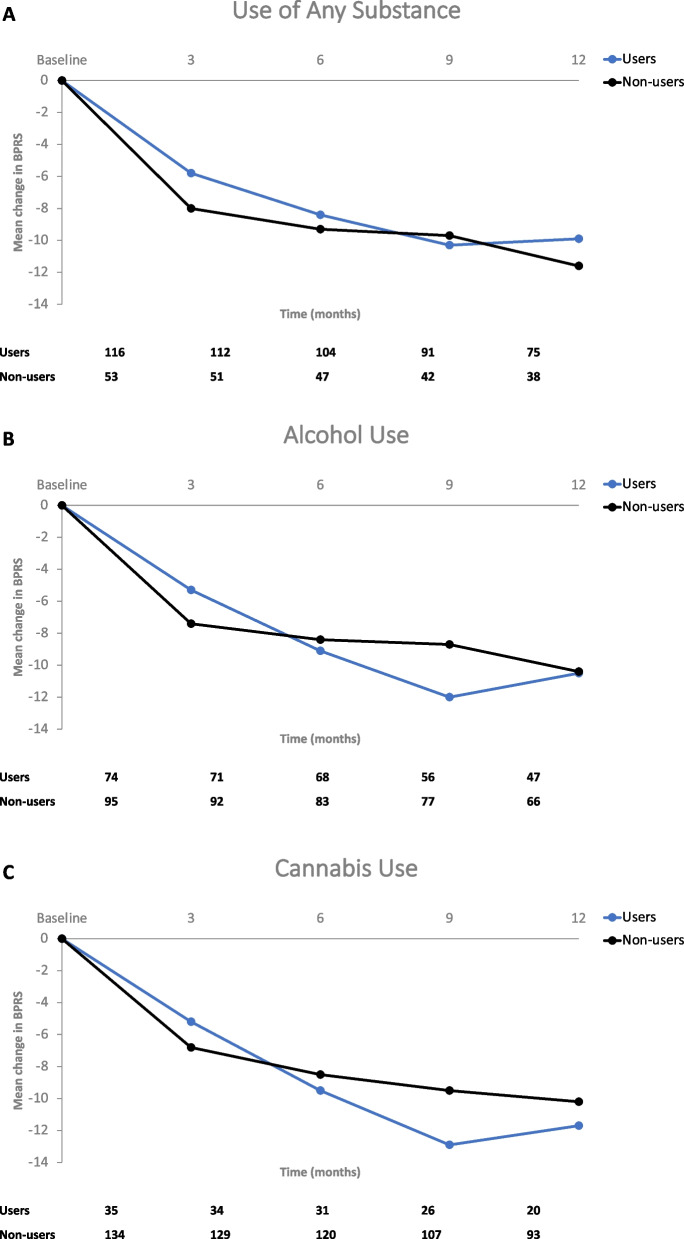
Change in Brief Psychiatric Rating Scale (BPRS) – Total Score over Time in Substance Users and Non-Users. **A** Patients reporting any substance use at baseline. **B** Patients reporting alcohol use at baseline. **C** Patients reporting cannabis use at baseline

Decrease from baseline in BPRS total scores after AOM treatment for 12 months showed no significant difference between users and non-users of any substances (-9.9 vs. -11.6, *P* = 0.441) (Fig. 4A), between alcohol users and non-users (-10.5 vs. -10.4, *P* = 0.967) (Fig. 4B), or between cannabis users and non-users (11.7 vs. -10.2, *P* = 0.578) (Fig. 4C).

### Adverse drug reactions

**Fig. 5:**
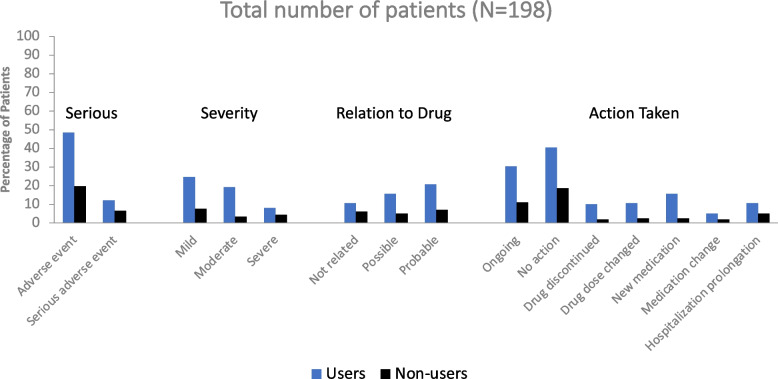
Adverse Drug Reactions by Substance Users and Non-Users

A total of 198 patients were evaluated in the safety analysis. Adverse drug reactions were primarily non-serious and mild and have been reported in detail in the primary publication [18]. However, in this *post hoc* analysis, the rate of occurrence of adverse events was notably higher amongst substance users vs. non-users (48.5% vs. 19.7%). This pattern was reflected across all safety parameters examined (Fig 5).

## Discussion

The results of this *post hoc* analysis of a real-world prospective cohort study show that treatment of schizophrenia with AOM for 12 months is as effective at improving global functioning in patients with concomitant substance use as in those without. Both groups demonstrated similar and significant improvements. It is important to make the distinction between patients in many studies cited herein who had formal diagnoses of SUD based on standardized criteria, and the patients in this *post hoc* analysis who simply self-reported use or non-use of substances.

Previous studies have reported on the impact of treatment with AOM in patients with psychosis and SUD. In a head-to-head comparison of AOM versus paliperidone LAI, quality of life and substance craving indicators improved in both groups, with greater effect in those treated with AOM [14]. More recently, a retrospective study by Szerman et al. (2020) reported on the efficacy and impact of AOM in patients with schizophrenia and comorbid substance use disorders [19]. The results showed that AOM reduced disease severity scores by >30% and significantly improved daily functioning in these patients. In this *post hoc* analysis, treatment with AOM also significantly improved outcomes in patients with reported substance use.

There are a few potential implications of the results of this *post hoc* analysis. A wide range of studies have demonstrated the association between substance use and poor medication adherence in patients with schizophrenia, leading to treatment discontinuation and relapse [20–22]. A systematic review of factors influencing medication adherence in individuals with schizophrenia identified substance abuse as a key driver of non-adherence [23]. In a Canadian cross-sectional survey of 207 psychiatric outpatients, those with a psychotic disorder and current substance use were more likely to be medication non-compliant than those with a single diagnosis of psychotic disorder (27.6% vs. 4.5%) [1]. LAI antipsychotic medications reduce adherence demands compared with daily oral antipsychotics and are a recommended treatment option for non-adherent patients [24]. A study of adherence with oral versus LAI antipsychotics in patients with early psychosis showed a significantly higher proportion of days with medication in the LAI formulation group than in those treated with oral antipsychotics (76% vs. 32%) [25]. This is important because effective early treatment with antipsychotic medication decreases relapse risk, leading to better outcomes [26]. A two-year cluster randomized clinical study showed that administration of a LAI medication (AOM) was associated with a delayed time to hospitalization [27]. Evidence from a systematic literature review supports the assertion that LAI antipsychotics are associated with enhanced adherence and reduced relapse rates, but the small number of available studies necessitate cautious interpretation of this result [28]. Real-world data of patients with schizophrenia showed significantly better medication adherence (33.9% vs. 25.5%) in patients receiving LAI antipsychotics compared to oral antipsychotic users [9]. The LAI group was also 20% less likely to discontinue their medication during the entire follow-up period [29]. In another real-world study comparing AOM with oral antipsychotics in patients with schizophrenia, individuals treated with AOM had significantly greater medication adherence and significantly longer time to treatment discontinuation [30]. In this *post hoc* analysis of patients with schizophrenia and concomitant substance use, we hypothesize that the LAI formulation appears to provide a key additional benefit in this patient population, reducing instability of treatment adherence related to substance use and leading to better outcomes. Furthermore, in real-life settings, lack of adherence to an injectable medication is more easily monitored by the healthcare team, resulting in timely interventions.

The results of this *post hoc* analysis also suggest potential benefits for illness severity and functional status, in that improved treatment adherence reduces the risk of relapse [23, 31, 32], thus improving the lifetime trajectory of schizophrenia. Generally, patients with schizophrenia and SUD have a higher rate of relapse due to poor adherence to treatment. Relapses disrupt remission and interfere with recovery. They are associated with re-hospitalization, treatment resistance, and loss of gains in function [33]. Subsequent episodes of relapse in a patient’s lifetime may render recovery more difficult due to disease progression and treatment refractoriness [34]. This is meaningful to clinicians because effective management of schizophrenia requires continuous long-term treatment. Therefore, treatment options that improve adherence should be prioritized regardless of whether patients will continue to use substances abuse or not. In addition, LAI’s have been shown to have small peak-to-trough fluctuations in drug levels which may increase their tolerability compared to oral therapeutics [35]. An increase in tolerability may in turn affect treatment adherence.

Finally, the observed lack of difference in global functioning between schizophrenia substance users and non-users may also reflect the level of patient deficits. Patients with schizophrenia who are also substance users may have fewer negative symptoms of schizophrenia, more social contacts, and better social-leisure functioning [36]. A meta-analysis highlighted the importance of intermediate factors such as the preferred substance used in understanding the contradictory cognitive capacities among substance-abusing patients with schizophrenia [37]. For example, preferential use of cannabis, as opposed to alcohol, by patients with schizophrenia and SUD was associated with higher scores for problem solving and reasoning, and visual memory. These cognitive features in patients with dual diagnosis may affect their capacity to be more structured and capable of the necessary socialization to go out to procure and use their substance.

### Limitations

Limitations related to the design of the ReLiAM study were previously described [18]. Although real-world evidence is more aligned with daily clinical practice than randomized clinical trials, its interpretation poses challenges related to the lack of a control group, potential selection bias, and data quality management. While this *post hoc* analysis informs on outcomes related to the subgroup of substance users that were not captured by the primary analysis, these results are also subject to limitations inherent in *post hoc* analyses. Possible interpretations are mostly limited to users of alcohol and cannabis, as the number of patients using other substances was too low to make any meaningful comparisons. Finally, data could not be analyzed beyond 12 months of treatment due to the 12-month study duration of the original ReLiAM study.

## Conclusion

This *post hoc* analysis of the real-world ReLiAM study demonstrates that treatment with AOM for 12 months in patients with schizophrenia was effective in improving global functioning in subgroups of patients with and without concomitant substance use. A lack of significant differences between substance users and non-users was also evident through multiple secondary endpoints measuring function and disease severity. Adverse events occurred more frequently in substance users vs. non-users; however, these were primarily non-serious and mild. These results support the use of AOM for the treatment of schizophrenia in patients with or without concomitant substance use. In light of evidence demonstrating improved adherence with AOM versus oral treatment in patients with comorbid schizophrenia and substance use, the AOM treatment paradigm may be particularly beneficial for this group of patients.

## Supplementary Information

**Additional file 1: Supplemental Figure 1.** Absolute Change from Baseline in Global Assessment of Functioning Scale GAF over Time in Heavy Substance Users and Non-Users of Substances. (A) Patients reporting heavy cannabis use at baseline. (B) Patients reporting heavy alcohol use at baseline.

## Data Availability

For ethical reasons, to ensure the privacy of the patient-level data utilized in the current study, and for reasons related to data ownership by the sponsor, data cannot be made available. However, data could be made available upon request for the purpose of conducting meta-analytic review in future.

## Abbreviations

ADR: Adverse Drug Reactions
AOM: Aripiprazole Once-monthly Injectable Formulation
BRPS: Brief Psychiatric Rating Scale
CGI-S: Clinical Global Impression Severity
GAF: Global Assessment of Functioning Scale
LAI: Long-Acting Injectable
ReLiAM: Real-Life Assessment of Abilify Maintena
SD: Standard Deviation
SOFAS: Social and Occupational Functioning Assessment Scale
SUD: Substance Use Disorder
