# Supplementary material for: Treatment with aripiprazole once-monthly injectable formulation is effective in improving symptoms and global functioning in schizophrenia with and without comorbid substance use – a post hoc analysis of the ReLiAM study
Source: BMC Psychiatry. 2022 Dec 8;22:773. doi: 10.1186/s12888-022-04397-x (PMC9733174; doi:10.1186/s12888-022-04397-x)
Supplement: Supplementary file 1 — Additional file 1: Supplemental Figure 1. Absolute Change from Baseline in Global Assessment of Functioning Scale GAF over Time in Heavy Substance Users and Non-Users of Substances. (A) Patients reporting heavy cannabis use at baseline. (B) Patients reporting heavy alcohol use at baseline. [file 12888_2022_4397_MOESM1_ESM.docx]

**Supplemental Figure 1**


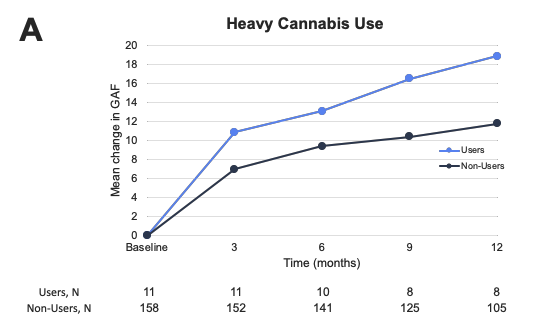


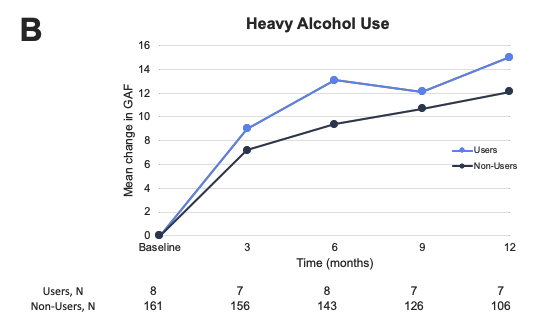


**Supplemental Figure 1: Absolute Change from Baseline in Global Assessment of Functioning Scale GAF over Time in Heavy Substance Users and Non-Users of Substances.** (A) Patients reporting heavy cannabis use at baseline. (B) Patients reporting heavy alcohol use at baseline.
